# NMR-Based Identification of Metabolites in Polar and Non-Polar Extracts of Avian Liver

**DOI:** 10.3390/metabo7040061

**Published:** 2017-11-16

**Authors:** Fariba Fathi, Antonio Brun, Katherine H. Rott, Paulo Falco Cobra, Marco Tonelli, Hamid R. Eghbalnia, Enrique Caviedes-Vidal, William H. Karasov, John L. Markley

**Affiliations:** 1 Biochemistry Department, University of Wisconsin-Madison, 433 Babcock Drive, Madison, WI 53706, USA; ffathi@wisc.edu (F.F.); paulo@nmrfam.wisc.edu (P.F.C.); mtonelli@wisc.edu (M.T.); hamid.eghbalnia@wisc.edu (H.R.E.); 2 Department of Forest and Wildlife Ecology, University of Wisconsin-Madison, 1630 Linden Drive, Madison, WI 53706, USA; abrun@wisc.edu (A.B.); krott@wisc.edu (K.H.R.); wkarasov@wisc.edu (W.H.K.); 3 Instituto Multidisciplinario de Investigaciones Biológicas de San Luis, Consejo Nacional de Investigaciones Científicas y Técnicas, and Departamento de Bioquímica y Ciencias Biológicas, Universidad Nacional de San Luis, San Luis 5700, Argentina; enrique.caviedesvidal@wisc.edu

**Keywords:** NMR spectroscopy, liver tissue, extraction protocol, metabolite identification by NMR, diet, effect of on liver metabolites

## Abstract

Metabolites present in liver provide important clues regarding the physiological state of an organism. The aim of this work was to evaluate a protocol for high-throughput NMR-based analysis of polar and non-polar metabolites from a small quantity of liver tissue. We extracted the tissue with a methanol/chloroform/water mixture and isolated the polar metabolites from the methanol/water layer and the non-polar metabolites from the chloroform layer. Following drying, we re-solubilized the fractions for analysis with a 600 MHz NMR spectrometer equipped with a 1.7 mm cryogenic probe. In order to evaluate the feasibility of this protocol for metabolomics studies, we analyzed the metabolic profile of livers from house sparrow (*Passer domesticus*) nestlings raised on two different diets: livers from 10 nestlings raised on a high protein diet (HP) for 4 d and livers from 12 nestlings raised on the HP diet for 3 d and then switched to a high carbohydrate diet (HC) for 1 d. The protocol enabled the detection of 52 polar and nine non-polar metabolites in ^1^H NMR spectra of the extracts. We analyzed the lipophilic metabolites by one-way ANOVA to assess statistically significant concentration differences between the two groups. The results of our studies demonstrate that the protocol described here can be exploited for high-throughput screening of small quantities of liver tissue (approx. 100 mg wet mass) obtainable from small animals.

## 1. Introduction

The liver, the most metabolically active organ, has multiple vital functions related to digestion, metabolism, and immunity. It processes the nutrients absorbed by the small intestine and produces bile that is released into the intestine for digesting fat. Therefore, liver has been called the body’s “chemical factory”. Owing to its important role in the digestive system, researchers have developed several methods to assay different liver fractions, including NMR-based metabolomics [1,2]. ^1^H NMR spectroscopy has been utilized since the 1970s to determine metabolic profiles in biological fluids and extracts [3]. NMR has several advantages over mass spectrometry for quantifying metabolite levels, including limited handling and preprocessing of biological samples, high reproducibility, non-destructive analysis, and high-throughput fingerprinting [4,5].

A crucial step for metabolomic studies of fluids is sample preparation. Several solvent systems for liver extraction have been investigated [6,7], and, from the various possibilities, we chose the methanol/chloroform/water solvent combination [8] for the current study. This solvent system yields NMR spectra of the polar fraction with improved baseline and linewidth, thanks to the exclusion of lipids and proteins from the aqueous phase. The chloroform phase contains non-polar metabolites.

The methanol/chloroform/water extraction method described here offers a rapid approach to simultaneous characterization of both the hydrophilic and hydrophobic metabolome. Because defects in lipid metabolism are known to be associated with diseases [9], finding a method to assay non-polar metabolites in liver can be very useful for functional and pathological studies of this tissue.

The Karasov laboratory is investigating the effects of diet on the metabolism of small (approx. 7–15 g) nestling birds. Our primary goal was to develop an NMR-based approach for identifying and quantifying the polar and non-polar metabolites present in liver tissue from these birds. Our secondary goal was to develop a database of metabolites from liver tissue that can be detected by NMR spectroscopy for use in future studies. To maximize the sensitivity of these sample-limited experiments, we made use of a 600 MHz NMR spectrometer equipped with a cryogenic 1.7 mm NMR probe, which enabled analysis of 70 µL extracts from 100 mg tissue. As reported here, our protocol enabled the detection of 52 polar and nine non-polar metabolites and revealed statistically different levels of non-polar metabolites in nestling birds fed on diets with either high carbohydrate or high protein but with identical lipid contents.

## 2. Results

**Figure 1:**
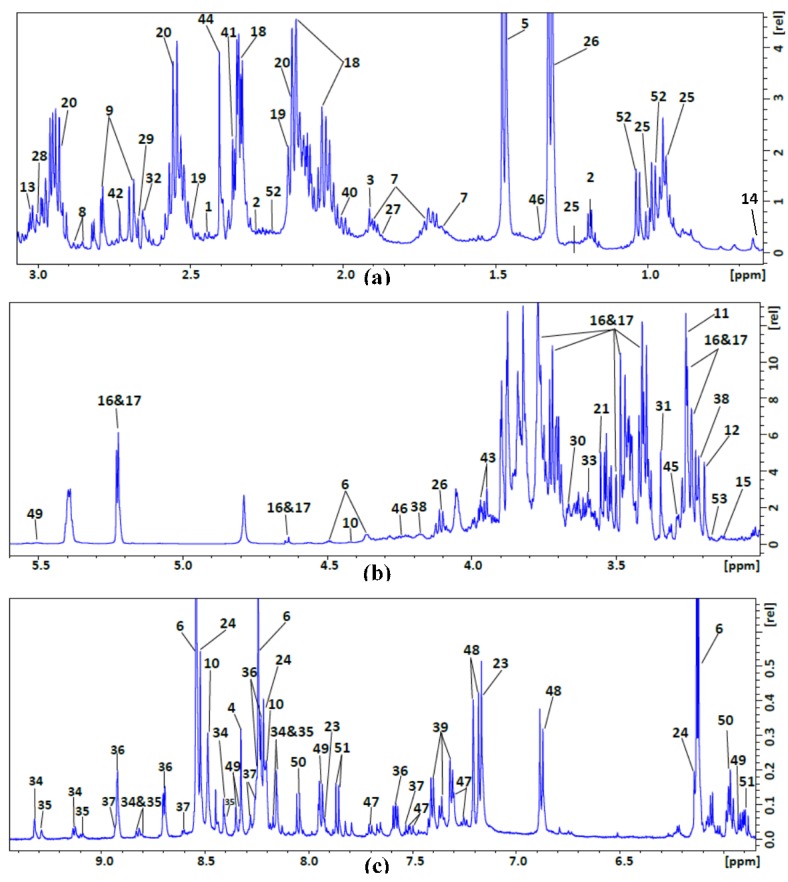
^1^H NMR spectra of the polar extract from avian liver. (**a**,**b**) Low frequency region. (**c**) High frequency region.

**Figure 2:**
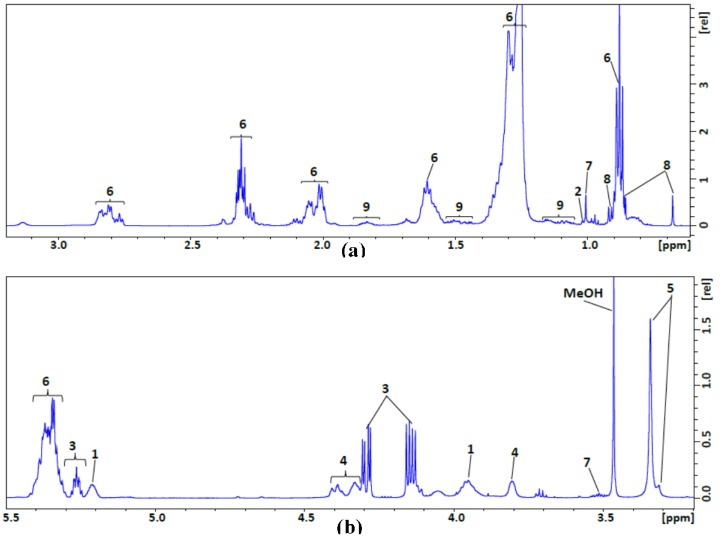
^1^H NMR spectra of the non-polar extract from avian liver. (**a**) Low frequency region. (**b**) High frequency region.

NMR data were collected on a 600 MHz NMR spectrometer equipped with a 1.7 mm cryogenic probe, which afforded the sensitivity needed for these small samples. Representative one-dimensional (1D) ^1^H NMR spectra of the polar and non-polar fractions from the liver tissue investigated in this study are presented in Figure 1 and Figure 2, respectively. We used peak intensities from assigned peaks in the ^1^H NMR spectra of individual samples to determine the metabolite concentrations with assistance of Chenomx NMR suite 8.2 software (Chenomx Inc., Edmonton, AB, Canada). The ^1^H NMR peak assignments to individual metabolites were verified and refined with reference to a 2D ^1^H,^13^C HSQC spectrum. To acquire the 2D ^1^H,^13^C HSQC spectrum (shown in Supplementary Materials Figure S1) in a reasonable amount of time, we combined the polar fractions from three birds. We assigned the peaks by comparing the chemical shifts of the 1D ^1^H and 2D ^1^H,^13^C HSQC NMR spectra with those from reference spectra deposited in the Biological Magnetic Resonance data Bank (BMRB) [10] and the Human Metabolome Database (HMDB) [11]. We were able to identify 52 metabolites present in the polar fraction and nine in the non-polar fraction.

Table 1 and Table 2 list the metabolites identified in the polar and nonpolar phases, respectively. In the polar fraction, we identified essential amino acids (valine, isoleucine, leucine, lysine, methionine, phenylalanine, threonine, and tryptophan), conditional amino acids (arginine, glutamine, tyrosine, glycine, proline, and serine), and nonessential amino acids (alanine, asparagine, and aspartate). The polar fraction contained metabolites from various pathways: β-alanine metabolic pathway (β-alanine, aspartate and histidine); glycine, serine, and threonine metabolism pathway (choline, threonine, betaine, sarcosine, creatine, glycine, serine, and pyruvate); glutathione metabolic pathway (glycine, glutathione, and NADP); nicotinate and nicotinamide metabolic pathway (NAD, NADP, niacinamide, and nicotinate); taurine and hypotaurine pathway (taurine); and alanine, aspartate and glutamate metabolic pathway (succinate, asparagine, glutamine, and 2-oxoglutarate). In addition, the spectra contain valuable information on nucleotides and cofactors, including ATP, GTP, AMP, IMP, UMP, NAD, and NADP+.

In the lipid fraction, 23 lipid-related resonances from nine forms of non-polar molecules were identified by analyzing our NMR spectra in light of literature data [9]. These include glycerophospholipid backbone, esterified cholesterol, glycerol backbone, phosphatidylcholine, sphingomyelin, fatty acyl chain, free cholesterol, total cholesterol and multiple cholesterol protons.

As can be seen from the ^1^H NMR spectrum of the non-polar fraction (Figure 2), several distinct signals from non-overlapping peaks can be assigned to portions of fatty acyl chains: methyl, methylene (–CH=CH–), allylic (=CHCH_2_–), olefinic (–CH=CH–), and diallylic (=CHCH_2_CH=) groups. As mentioned above, lipids or fats play an important role in biological function in the body; among these are simple lipids or homolipids, compound lipids or heterolipids and derived lipids. Among triglycerides, triacylglycerols can be identified on the basis of their distinctive ^1^H NMR peak at 4.15 ppm. The sum of LDL (low-density lipoprotein) cholesterol, HDL (high-density lipoprotein) cholesterol, and VLDL (very low-density lipoprotein) is called “total cholesterol”. Cholesterol also plays a critical role as the precursor to many biologically important substances, including bile acids. Total cholesterol (C-18 H_3_) can be measured from the ^1^H NMR peak at 0.68 ppm.

**Figure 3:**
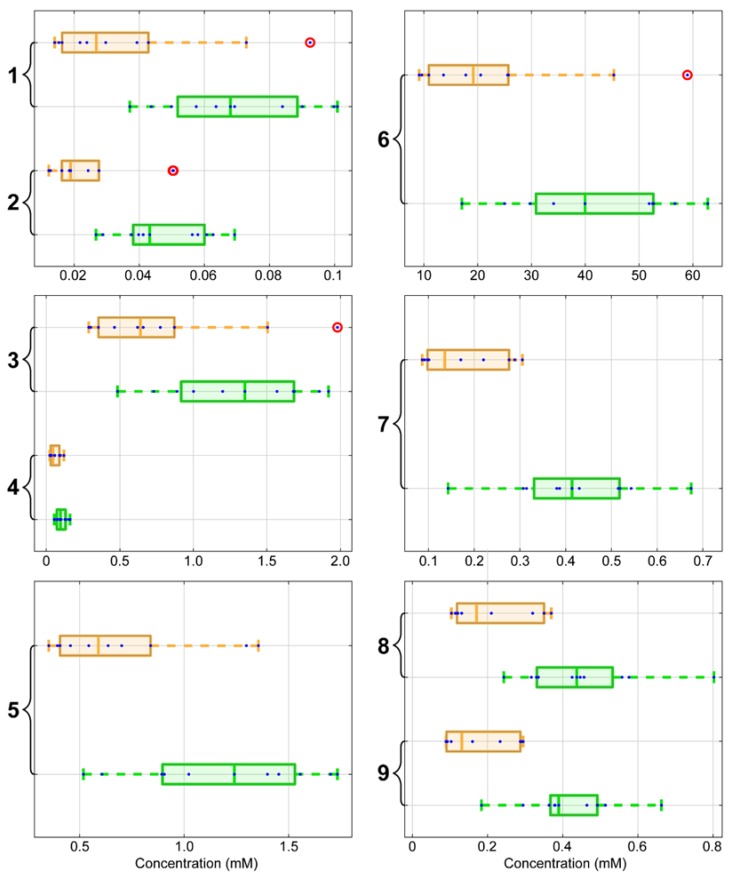
Box plots for the nine metabolites found non-polar extracts from liver of birds from group 1 fed on a high carbohydrate diet (orange) and group 2 fed on a high protein diet (green). The numbers correspond to the peak labels in Figure 2 and Table 2: 1 = glycerophospholipid; 2 = esterified cholesterol; 3 = glycerol backbone; 4 = phosphatidyl choline; 5 = sphingomyelin and choline; 6 = fatty acyl chain; 7 = free cholesterol; 8 = total cholesterol; and 9 = multiple cholesterol protons. The *x*-axis represents concentration in mM. The vertical line within each box represents the median, and the box limits indicate the first and third percentiles. The difference between the two box limits is the interquartile range or *IQR*. Data points are represented by blue dots. Suspected outliers (circled in red) lie outside the whiskers shown at 1.5 × *IQR* above the third quartile and 1.5 × *IQR* below the first quartile.

To evaluate the protocol described here, we analyzed differences in the metabolic profile of liver tissue from 7 d old house sparrow fed with two different diets. Nestlings were raised on either a HP diet for 4 d (group 2 in in Figure 3) or were raised on a HP diet for 3 d and then switched to a HC diet for 1 d (group 1 in Figure 3). We found clear differences in the concentrations of non-polar metabolites in the two groups as shown by the box plots in Figure 3. One-way ANOVA analysis (Supplementary Materials) shows that several metabolites were significantly different between the two groups at the 0.05 significance level: glycerophospholipid backbone, esterified cholesterol, glycerol backbone, multiple cholesterol protons, free cholesterol, total cholesterol, fatty acyl chain. Three others were not significantly different: phosphatidyl choline, sphingomyelin, and choline N(CH3)3d. These differences are of interest because the lipid compositions of the diets fed to the two groups was identical.

## 3. Discussion

We have evaluated a simple protocol for simultaneously characterizing the lipidic and hydrophilic metabolic profiles in liver tissue of nestling birds. We used NMR spectroscopy to identify and quantify 61 polar and non-polar metabolites through an array of 1D and 2D NMR experiments. Our procedure enabled us to identify and confirm a larger number of metabolites from liver tissue than previous NMR-based investigations [12,13,14,15,16,17]. Three of the earlier studies also used 100 mg samples of liver tissue [14,15,16], and three also prepared polar and non-polar extracts from the same samples [15,16,17]. From this comparison, we conclude the advantages stem from our use of more concentrated extracts in a 1.7 mm cryogenic probe, which accepts smaller samples than a 5 mm probe (40 µL rather than 300–500 µL).

The extraction of liver tissue with methanol/chloroform/water has the advantage of liberating bound metabolites from denatured protein. The disadvantage is that the concentrations of some metabolites may have changed in the period between the time that the animals were sacrificed and the liver tissue flash frozen. The high average glucose (189 mM) to lactate (40 mM) ratio suggests the absence of acidosis in the tissue samples.

We have shown that the protocol has the potential of identifying and quantifying differences in liver metabolites between groups of nestling birds raised on different diets. This procedure for high-throughput identification of 61 metabolites in liver should be useful for biological research where sample quantities are limited. To encourage replication of our results and their analysis by future improved methods, we have deposited all primary NMR data from this study in the BMRB archive [10] under accession bmme000001.

## 4. Materials and Methods

### 4.1. Animal Model 

Nestling house sparrows (*Passer domesticus*) were collected 3 d post hatch from nests on the campus of University of Wisconsin-Madison between mid-May and early August of 2016 using methods described earlier [18]. In the laboratory, they were syringe-fed hourly 15 times per day for 4 d in total. Two synthetic liquefied diets were used. The high protein diet or HP was 59.5% casein and 5% corn starch, and it was designed to represent a natural diet composed of mainly arthropods that wild house sparrow nestlings consume during the first few days post-hatch. The high carbohydrate diet or HC was 26.5% casein and 38% corn starch, and it was designed to represent the combination of arthropods and seeds that is consumed by wild fledgling house sparrows. Both diets contained 8% corn oil. Nestlings on both diets were fed the same quantities of gross energy per day (verified by mass, ±0.01 g), although meal sizes were gradually increased as they grew. The birds were randomly assigned to two different groups. In one group, the “high protein diet” group (group 2 in Figure 3, the nestlings were fed 4 d with the HP diet. In the other group, the “high carbohydrate diet” group (group 1 in Figure 3), nestlings were fed with the HP diet for 3 d and 1 d with the HC diet. Livers from the high protein diet group had about 17% greater mass than those from the high carbohydrate diet group. All experimental procedures were approved by the University of Wisconsin, Madison, Ethics Committee (permit no. RARC A-0570-0-03-14).

### 4.2. Sample Preparation 

Nestlings were euthanized with CO_2_, and dissected to remove the intestine, pancreas, and liver. The whole liver was placed in ice-cold Hanks’ balanced salt solution-mannitol and then blotted dry, divided in two, and weighted. The part used for this analysis was frozen immediately in liquid nitrogen and stored at −80 °C until metabolite extraction by the method described by Beckonert et al. [8]. An amount of 100 mg of intact frozen tissue in 400 µL ice-cold methanol and 85 µL ice-cold water was homogenized on ice using an Omni 5000 homogenizer (Omni International, Waterbury, CT, USA) by 30 s pulses over a period of 3 min. After vortexing for 10 s, 200 µL cold chloroform was added, and the sample was vortexed again for 30 s. The mixture was incubated on ice for 15 min and then centrifuged at 1000× *g* for 15 min at 4 °C. This procedure generated three phases. The polar metabolites in the methanol/water phase at the top are separated from the lipophilic part in the chloroform phase at the bottom by a thin layer that includes proteins, nucleic acids, and cellular debris. This layer between the two phases was discarded, while the methanol/water and chloroform layers were kept for further analysis. Solvent from each phase was removed from the samples with a speed vacuum concentrator [8].

### 4.3. NMR Analysis

The dried non-polar fractions were dissolved in 40 µL deuterated chloroform-TMS (chloroform-D (99.8% D) + 0.05% (*v*/*v*) TMS). The dried polar fractions were solubilized by adding 40 µL of buffer (100 mM sodium phosphate buffer, pH 7.4, in D_2_O, containing 0.1 mM DSS and 0.4% NaN_3_). The solutions were then transferred to 1.7 mm NMR tubes and kept at −20 °C prior to acquiring NMR spectra. All spectra were recorded at 298 K on a Bruker Advance III 600 MHz spectrometer (operating at 600.08 MHz for ^1^H) equipped with a 1.7 mm cryogenic probe. The ^1^H NMR water signal from the polar fraction was suppressed by means of excitation sculpting (Bruker ZGESGP pulse program). ^1^H NMR spectra of the non-polar fraction were acquired with the ZG30 pulse sequence (Bruker BioSpin, Billerica, MA, USA). All ^1^H NMR spectra were the average of 256 transients acquired with 32,768 points, an acquisition time (AQ) of 1.70 s, and a repetition delay of 1 s between transients. The chemical shifts of the polar and non-polar fractions were referenced to DSS and TMS, respectively. Relative concentrations of metabolites in the non-polar fraction were measured from peak areas relative to that from the chloroform peak. Spectra were manually phased and baseline-corrected using Bruker Topspin 3.5 software (Bruker). To assist in identifying the metabolites, two-dimensional ^1^H-^13^C heteronuclear single quantum coherence (HSQC) spectra were acquired using a gradient selected, sensitivity enhanced pulse program. To optimize the sensitivity of these experiments, the polar extracts from three samples were combined and concentrated to the volume of a single NMR sample. Each time-domain spectrum of the HSQC experiment was the average of 64 transients consisting of 4096 points with a 1-s repetition delay; the second dimension was derived from 256 increments. The spectral widths were 16 ppm and 175 ppm for the ^1^H and ^13^C dimensions, respectively.

## Figures and Tables

**Table 1:** Compounds identified in the polar extract of avian liver tissue from NMR data: peak numbers (as shown in Figure 1a,b) and assigned chemical shifts (multiplicities).

No.	Compound Name	^1^H NMR Chemical Shift (ppm), Multiplicity
1	2-Oxoglutarate	2.4 t, 3 t
2	3-Hydroxybutyrate	1.2 d, 2.3 m, 2.4 m, 4.1 m
3	Acetate	1.9 s
4	Adenosine	8.3 s, 8.2 s, 6.1 d, 4.8 t, 4.4 m, 4.3 m, 3.9 dd, 3.8 dd
5	Alanine	1.47 d, 3.78 q
6	AMP	4.01 m, 4.36 m, 4.50 q, 4.79 t, 6.12 d, 8.25 s, 8.58 s
7	Arginine	1.66 m, 1.91 m, 3.27 t, 3.77 t
8	Asparagine	2.85 dd, 2.94 dd, 4.00 dd
9	Aspartate	2.66 dd, 2.80 dd, 3.91 dd
10	ATP	8.49 s, 8.24 s, 6.12 d, 4.73 t, 4.56 t, 4.27 m, 4.23 m
11	Betaine	3.25 s, 3.88 s
12	Choline	3.19 s, 3.50 dd, 4.05 t
13	Creatine	3.02 s, 3.91 s
14	DSS	0.00 s, 0.62 t, 1.75 m, 2.90 m
15	Ethanolamine	3.13 t, 3.83 t
16	α-Glucose	3.42 m, 3.54 m, 3.72 m, 3.73 m, 3.77 m, 3.87 m, 5.23 d
17	β-Glucose	3.25 m, 3.49 m, 3.49 m, 3.50 m, 3.88 m, 3.91 m, 4.66 d
18	Glutamate	2.04 m, 2.12 m, 2.32 m, 2.32 m, 3.76 dd
19	Glutamine	2.15 m, 2.18 m, 2.42 m, 2.46 m, 3.76 t
20	Glutathione	2.17 m, 2.50 m, 2.56 m, 2.94 dd, 2.98 dd, 3.83 m, 4.56 q
21	Glycine	3.54 s
22	GTP	8.13 s, 5.93 d, 4.74 t, 4.54 t, 4.35 m, 4.22 m, 4.25 m
23	Histidine	3.16 dd, 3.23 dd, 3.98 dd 7.11 s, 7.86 s
24	IMP	8.53 s, 8.21 s, 6.13 d, 4.49 t, 4.36 m, 4.03 m
25	Isoleucine	0.93 t, 1.02 d, CH2 1.26 m, 1.46 dd, 1.97 m, 3.66 d
26	Lactate	1.31 d, 4.10 q
27	Leucine	0.94 d, 0.95 d, 1.71 m, 3.73 m
28	Lysine	1.43 m, 1.49 m, 1.71 m 1.87 m, 1.91 m, 3.01 t, 3.7 t
29	Malate	2.36 dd, 2.66 dd, 4.30 dd
30	Mannitol	3.66 m, 3.75 m, 3.80 d, 3.86 dd
31	Methanol	3.33 s
32	Methionine	2.11 s ,2.12 s, 2.19 m, 2.63 t, 3.85 t
33	Myo-inositol	3.26 t, 3.51 dd, 3.60 t, 4.04 t
34	NAD+	9.3 s, 9.13 d, 8.84 d, 8.42 s, 8.20 t, 8.16 s, 8.08 d,6.02 d, 4.75 t, 4.53 t, 4.49 m, 4.48 t, 4.36 m, 4.2 m
35	NADP+	9.28 s, 9.09 d, 8.81 d, 8.40 s, 8.18 m, 8.14 s, 6.09 d, 6.04 d, 4.97 m, 4.60 m, 4.49 m, 4.45 t, 4.39 q , 4.36 m, 4.31 m, 4.27 m, 4.2 m
36	Niacinamide	8.92 d, 8.70 d, 8.25 d, 7.58 q
37	Nicotinate	8.92 d, 8.59 d, 8.24 d, 7.51 q
38	*O*-Phosphocholine	3.21 s, 3.58 m, 4.16 m
39	Phenylalanine	3.11 dd, 3.28 dd, 3.99 dd, 7.31 m, 7.36 m, 7.41 m
40	Proline	1.97 m, 2.03 m, 2.33 m, 3.33 m, 3.41 m, 4.12 dd
41	Pyruvate	2.35 s
42	Sarcosine	2.72 s, 3.60 s
43	Serine	3.83 dd, 3.93 dd, 3.97 dd
44	Succinate	2.39 s
45	Taurine	3.26 t, 3.43 t
46	Threonine	1.32 d, 3.57 d, 4.25 m
47	Tryptophan	3.30 dd, 3.48 dd, 4.05 dd, 7.19 t, 7.27 t, 7.30 s, 7.52 d, 7.72 d
48	Tyrosine	3.04 dd, 3.19 dd, 3.93 dd, 6.88 m, 7.18 m
49	UDP-*N*-Acetylglucosamine	2.06 s, 3.55 t, 3.80 m, 3.87 m, 3.91 d, 3.98 t, 4.17 m, 4.23 m, 4.27 m, 4.35 m, 5.5 dd, 5.96 d, 5.98 d, 7.94 d, 8.34 d
50	UMP	3.96 d, 4.03 d, 4.25 s, 4.34 t, 4.41 t, 5.97 d, 5.98 d, 8.08 d
51	Uridine	3.80 dd, 3.90 dd, 4.12 dt, 4.21 dd, 4.34 dd, 5.89 d, 5.90 m, 7.86 d
52	Valine	0.98 d, 1.03 d, 2.26 m, 3.60 d
53	β-Alanine	2.53 t, 3.17 t

**Table 2:** Compounds identified in the non-polar extract of avian liver tissue from NMR data: peak numbers (as shown in Figure 2) and assigned chemical shifts (multiplicities).

No.	Compound Name	Assignment, ^1^H NMR Chemical Shift (ppm), Multiplicity
1	Glycerophospholipid backbone	C-3 H2 (3.96, s); C-2 H (5.17–5.24, m)
2	Esterified cholesterol	C-19 H3 (1.02, s)
3	Glycerol backbone	C-1 H2/C-3 H2 (4.15/4.29, m); C-2 H (5.26, p)
4	Phosphatidyl choline	N-CH2 (3.81, s-broad); PO-CH2 (4.32–4.43, m)
5	Sphingomyelin and choline	N(CH3)3 (3.32/3.35, s/s)
6	Fatty acyl chain	CH3(CH2)n (0.88, t); (CH2)n (1.24–1.37, m); –CH2CH2CO (1.55–1.65, m); –CH2CH= (1.98–2.09, m); –CH2CO (2.24–2.35, m); =CHCH2CH= (2.77–2.87, m); –HC=CH– (5.29–5.43,m)
7	Free cholesterol	C-19 H3(1.01, s); C-3 H(3.48–3.57, m)
8	Total cholesterol	C-18 H3( 0.68, s); C-26 H3/C-27 H3 (0.86/0.87, d/d); C-21 H3 (0.91, d)
9	Multiple cholesterol protons	1.05–1.19; 1.42–1.55; 1.79–1.88

## Supplementary Materials

The following are available online at www.mdpi.com/2218-1989/7/4/61/s1, Figure S1. **^1^**H,^13^C HSQC spectrum of the polar extract from avian liver: (a) methyl region, (b) sugar region, (c) aromatic region. One-way ANOVA analysis of the concentrations of the non-polar metabolites in extract from birds fed on the two different diets.
