# Supplementary material for: NMR-Based Identification of Metabolites in Polar and Non-Polar Extracts of Avian Liver
Source: Metabolites. 2017 Nov 16;7(4):61. doi: 10.3390/metabo7040061 (PMC5746741; doi:10.3390/metabo7040061)
Supplement: Supplementary file 1 [file metabolites-07-00061-s001.pdf]

Supplementary Materials

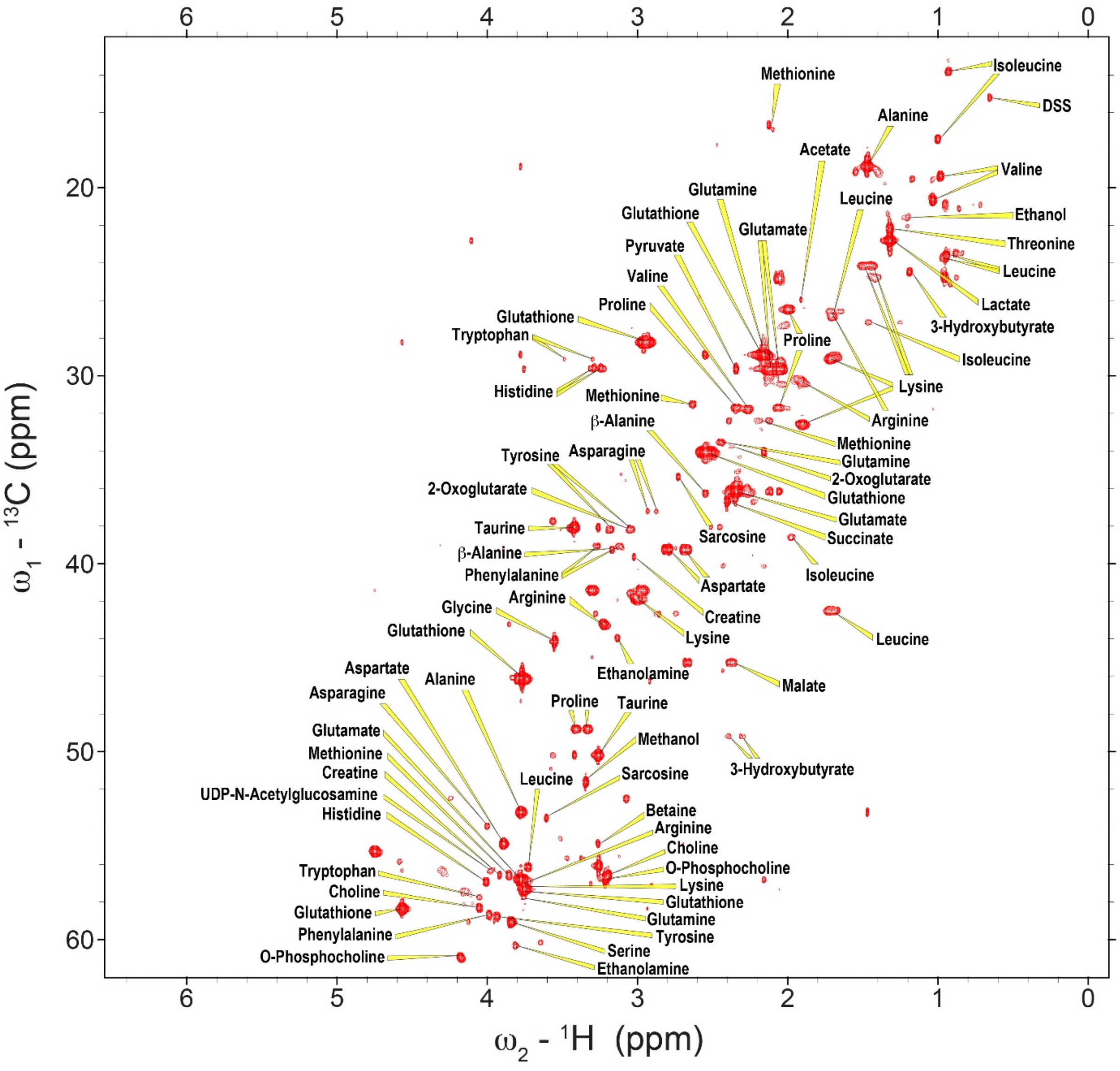

(a)

Figure S1. $^{1}H,^{13}C$ HSQC spectrum of the polar extract from avian liver: (a) methyl region.

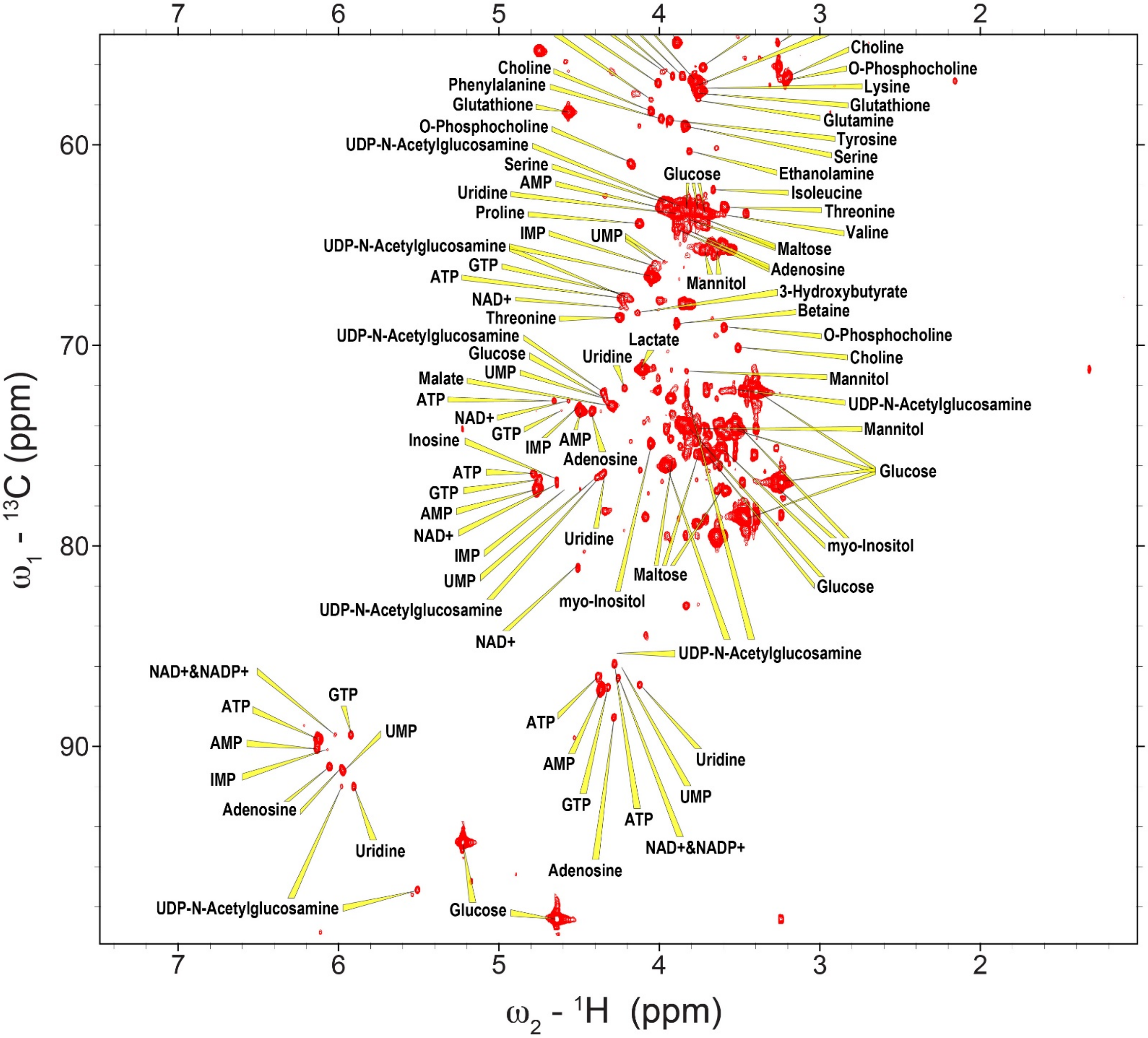

(b)

Figure S1. ^{1}H,^{13}C HSQC spectrum of the polar extract from avian liver: (b) sugar region.

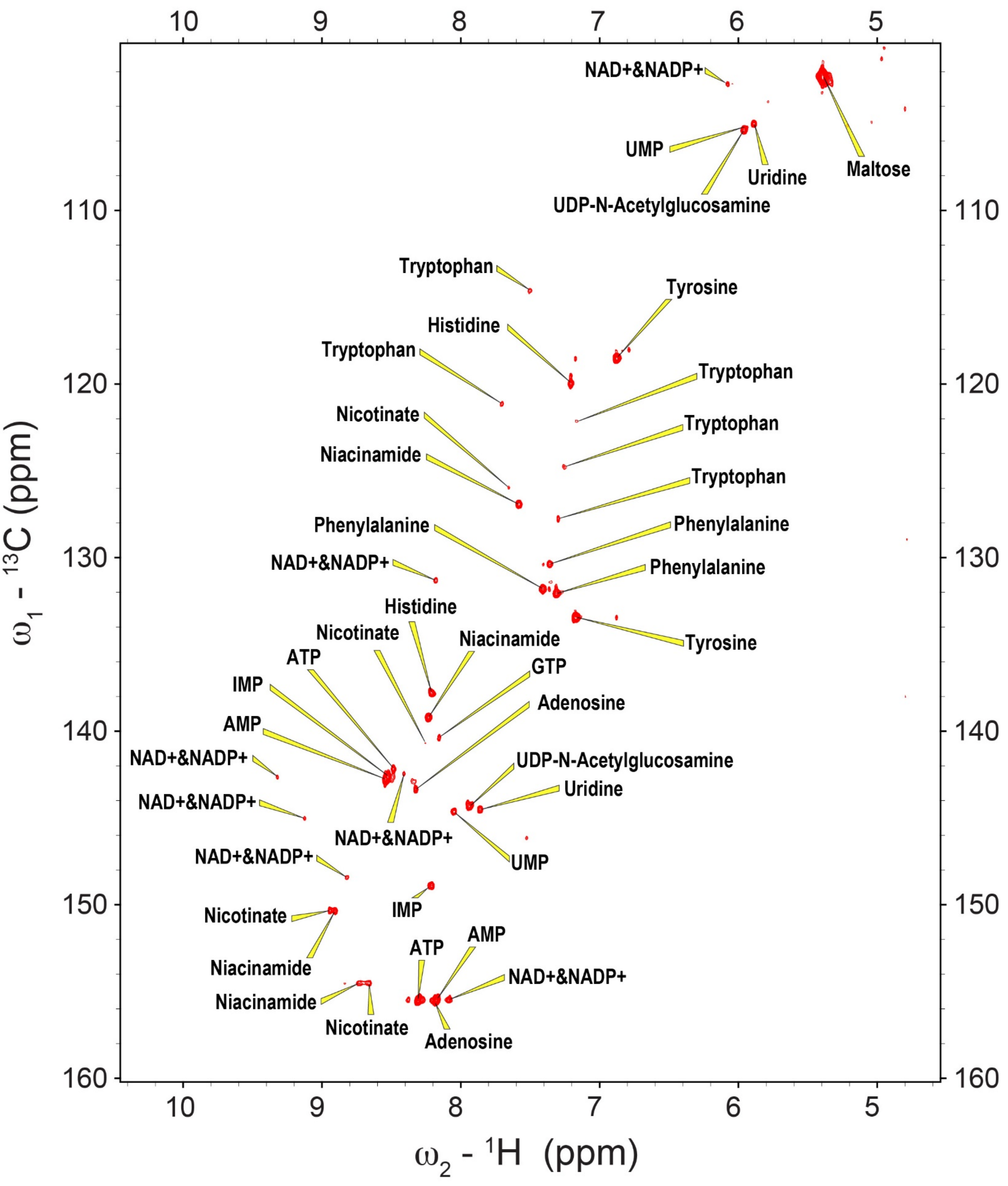

(c)

Figure S1. ^{1}H,^{13}C HSQC spectrum of the polar extract from avian liver: (c) aromatic region.

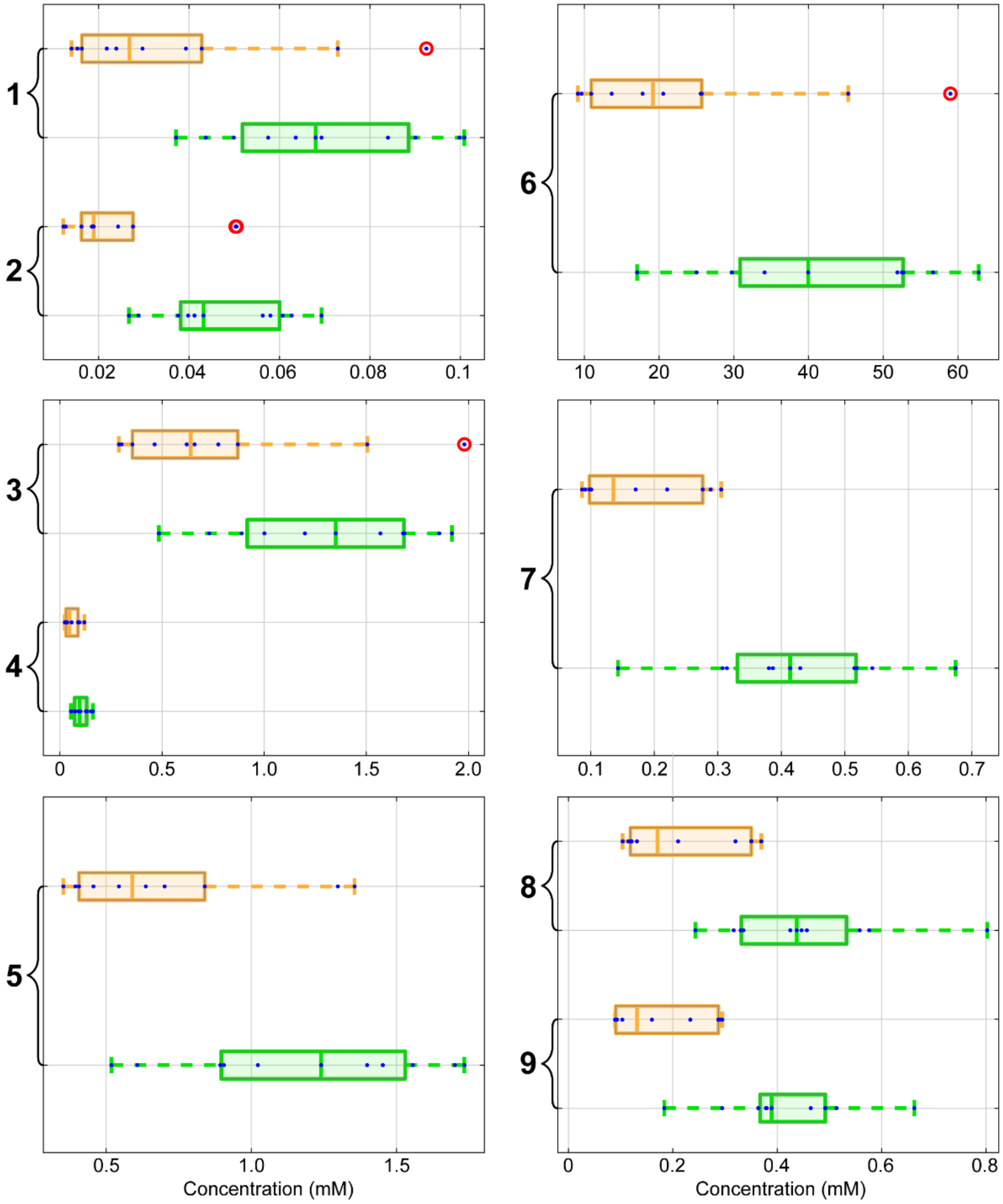

Figure S2. Box plots for the nine metabolites found non-polar extracts from liver of birds from group 1 fed on a high carbohydrate diet (orange) and group 2 fed on a high protein diet (green). The *x*-axis represents concentration in mM. The vertical line within each box represents the median, and the box limits indicate the first and third percentiles. The difference between the two box limits is the interquartile range or *IQR*. Data points are represented by blue dots. Suspected outliers (circled in red) lie outside the whiskers shown at 1.5×*IQR* above the third quartile and 1.5×*IQR* below the first quartile. The numbers agree with peak labels in Figure 2 and Table 2: 1 = glycerophospholipid; 2 = esterified cholesterol; 3 = glycerol backbone; 4 = phosphatidyl choline; 5 = sphingomyelin and choline; 6 = fatty acyl chain; 7 = free cholesterol;8 = total cholesterol; and 9 = multiple cholesterol protons.
